# Predicting the intention and adoption of wearable payment devices using hybrid SEM-neural network analysis

**DOI:** 10.1038/s41598-023-38333-0

**Published:** 2023-07-11

**Authors:** Abdullah Al Mamun, Farzana Naznen, Marvello Yang, Qing Yang, Mengling Wu, Mohammad Masukujjaman

**Affiliations:** 1 UKM-Graduate School of Business, Universiti Kebangsaan Malaysia, 43600 UKM Bangi, Selangor Darul Ehsan Malaysia; 2 UCSI Graduate Business School, UCSI University, Cheras, 56000 Kuala Lumpur, Malaysia; 3 Faculty Economic and Business, Widya Dharma University Pontianak, Pontianak, Kalimantan Barat 78243 Indonesia

**Keywords:** Psychology, Human behaviour

## Abstract

This study aims to examine the mediating effect of the intention to use wearable payment devices (WPD) between perceived ease of use (PE), perceived usefulness (PU), social influence (SI), perceived trust (TR), and lifestyle compatibility (CM) on the adoption of WPD. Examination was made on the moderating effect of age and gender to improve the understanding of the adoption of WPD as a new payment system. Empirical data was collected through an online survey from 1094 respondents in Malaysia. Furthermore, this study employed dual-stage data analysis through partial least squares structural equation modelling (PLS-SEM) to test the causal and moderating effects, including artificial neural network (ANN) to examine the predictive power of the selected model. As a result, it was found that PE, PU, TR, and CM had a significant positive influence on the intention to use WPD. Furthermore, facilitating conditions and the intention to use WPD exhibited strong positive impacts on the adoption of WPD among Malaysian youth. The intention to use WPD positively and significantly mediated all predictors of adoption of WPD. Following that, ANN analysis confirmed high prediction accuracy of the data fitness. Overall, the findings for ANN highlighted the importance of PE, CM, and TR on the intention to adopt WPD and the impact of facilitating conditions on the adoption of WPD among Malaysian youth. Theoretically, the study extended UTAUT with two additional determinants (e.g., perceived trust and lifestyle compatibility), which were found to have significant influences on the intention to use WPD. The study results would be able to help payment service providers and the smart wearable device industry offer an innovative spectrum of products and present effective marketing tactics to encourage the prospective consumers of Wearable Payment Devices in Malaysia.

## Introduction

Numerous emerging economic developments are at the edge of a digital revolution to build a payment system to ease the evolution from the cash-based method to a cashless system by crafting secure and trustworthy networks, flawless transaction solutions, and guaranteed large volume transactions at a higher speed. The widespread adoption of mobile devices including smartphones, Internet of Things, and smart wearable devices (e.g., smartwatches, wrist-band, and rings) has transformed conventional methods of payments from cash and card-based transactions to mobile payment systems^1,2^. Cashless payment or contactless payment denotes a smart payment option used by several developing countries^3^, which includes internet banking, mobile payment, near field communication (NFC) m-payment, e-wallet, and wearable payment devices (WPD). Through the rapid development of Internet of Things (IoT), payment technology currently offers the opportunity to extend payment capabilities beyond cards and mobile phones to a larger and diverse system of internet-connected wearable payment devices^1^. Additionally, the concept of integrating micropayments into wearable devices has become a topic of debate in the wearable device market^4^.

Wearable devices or technologies refer to portable and small electronic devices, which are connected to wireless systems or internet and integrated into gadgets, jewellery, accessories, and clothes that could be fixed on the body or used as micro-chips and smart tattoos^5^. Wearable payment refers to a contactless payment method made through wearable devices that support the NFC method^6^. A few prominent brands, such as Apple-Pay and Android-Pay, have presented several potential wearable payment systems, which are yet to achieve a noticeable market. Moreover, Samsung-Pay and Maybank Visa Pay-Band were also introduced in the Malaysian market as contactless payment options^1^. Despite the reality that wearable payment has a low market penetration rate^7^, it is expected to create new business opportunities in various product categories^1^. Wearable devices are among the most important IoT items in the global market with a significant increase in sales of approximately 141 million units in 2019. This increase doubled the total sale in 2017^8^. The recent forecasts on smart wearable devices stated that the sales of IoT-based and smart wearable devices could reach 57 billion USD by 2022, 64 billion USD by 2025, and 104 billion USD by 2027^9^. Based on other surveys of contactless payment systems conducted in 2019, 67% of customers were engaged in an m-payment transaction with 19% growth in Thailand, 24% growth in Vietnam, and 20% growth in Middle East countries^10^. Hence, with the consolidation of statistics of the rise in usage of smart wearable devices and the usage of contactless payments, a high prospect of wearable payment devices could be presumed in near future. Until recently, the wearable payment system is an exceptionally new concept, which requires thorough exploration about its adoption among Malaysian users.

Globally, there have been several research studies conducted on the adoption of WPD. Luyao et al.^11^ conducted a study in the context of China, which was limited to examining the direct relationship to adoption and did not consider mediation and moderation relationships in their empirical investigations. Rehman et al.^12^ investigated the factors affecting the adoption of smart wearable payment from a Saudi Arabian perspective, adopting an old model like TAM. They only focused on technological factors and overlooked external factors such as social influence. Rabaa'i and Zhu^13^ conducted research on similar adoption factors in Kuwait, integrating trust, but they did not consider external factors and indirect relationships (mediation and moderation) in their research framework. However, few empirical studies based on mobile payment usage^14,15^ and the adoption of wearable technologies^16^ were conducted in Malaysia. However, given that these studies mainly focused on m-payment methods and the adoption of wearable technologies, wearable payment devices were not taken into account. Considering the difference between the devices and procedures utilized in m-payment and wearable payments, the studies based on m-payment should not characterize similar behaviour that was perceived in the adoption of wearable payment devices^1^. Although the study by Lee et al.^1^ utilized an advanced analysis method (dual-stage SEM-ANN using deep learning), it had the limitation of not considering any external factors in their research framework. They only focused on cognitive factors and technology factors. Likewise, Hayet et al.^17^ using fuzzy set qualitative comparative analysis studied adoption factor of WPD in Malaysian perspectives, but and failed to use the indirect relationships. Chuah et al.^16^ researched on WPD adoption in Malaysia incorporating the control variable like age and gender, but did not used new constructs other than TAM and ignored to stress on any particular generation (young generation) cohort. Determining consumers’ intent of using new technology is the first step in examining its ultimate adoption. Previous studies focused on behavioural intention models and theories, including the Technology Acceptance Model (TAM)^18^, Diffusion of Innovation Theory (DOI)^19^, Unified Theory of Acceptance and Use of Technology (UTAUT)^14,20,21^, and UTAUT2^22^ for the investigation on the intention of using m-payment. The only study conducted in Malaysia on the behavioural intention towards wearable payment devices was the study by Lee et al.^1^, which adopted the MTAM and examined the influencing actors such as Optimism, Innovation, Discomfort, Insecurity, and Perceived Aesthetics. Considering the scarcity of research, this study adopted UTAUT to investigate the impacting factors (perceived ease of use, perceived usefulness, social influence, facilitating conditions) on the intention of using the wearable payment device among Malaysian youth. The adoption of any new technology is inclined by privacy concerns, perceived risk, and trust, although these factors have not been identified by both UTAUT and TAM^23^. Thus, these two constructs were adopted in this study to examine their impact on behavioural intention on wearable payment devices.

The young generation is considered advanced level consumers of internet and mobile devices, and also the prompt adopter of new technologies^24,25^. Compared to the younger consumers, senior consumers are perceived to be slower in accepting new technology and prefer face-to-face banking and financial services^26^. As a result, youths are the ideal population for all studies that employ new technical advancements^27^. Likewise, age and gender as moderators are two key variables in diverse research, such as mobile payment^18^, cashless payment^28^, and gamification^29^. These studies have shown gender and age-based differences in technology acceptance, making it important to investigate their role in shaping the relationships between the key variables^16^. Gender and age are known to be demographic variables that can significantly influence individuals' attitudes and behaviors, including technology adoption^30^. Understanding how gender and age may moderate the relationships in this study provides insights into potential variations in the adoption of WPD among different demographic groups^29^. Although the literature demonstrates that men present a higher degree of technology adoption than women^31^ and that young people present a higher degree of adoption than older users^32^, it is still unknown whether the direct relationships differ significantly in different genders or age groups for the adoption of WPD. An in-depth study including age and gender can provide insights for designing targeted strategies and interventions tailored to specific demographic segments. Therefore, the study aims to answer the following questions based on theoretical analysis: (a) among the potential predictors, which are the crucial determinants that influence the intention and ultimate usage of wearable devices for payment? (b) are factors of UTAUT adequate to explain all the influencing determinants, or are external constructs such as Lifestyle Compatibility and Perceived Trust also important antecedents? (c) what are the impacts of gender and age as moderators on the intention to use wearable payment devices?

To address the research questions, this study conducts an empirical study using a cross-sectional survey design among Malaysian youth. This study is significant because it applies the UTAUT framework by extending it with additional determinants of intention to use WPD (perceived trust and lifestyle compatibility). Furthermore, this study addresses the research gaps in the scarce literature on these factors in the context of WPD. Additionally, the study builds upon previous research on contactless payment methods and introduces WPD as a new form of payment, thereby adding to the existing body of knowledge. The incorporation of a two-step analysis methodology (SEM-ANN) provides valuable insights into the influences of key variables and demonstrates the accuracy of data fitness through neural network analysis. The findings emphasize the need for user-friendly and simplified WPD systems, trustworthiness, and privacy measures, as well as uninterrupted service, offering practical guidance to manufacturers and marketing practitioners. Overall, this study contributes to the understanding of WPD adoption and provides valuable insights for researchers and industry professionals in the Malaysian context and beyond.

The paper comprises multiple sections. The first section introduces the topic and provides an overview of the research. In the second section, a theoretical foundation is established, leading to the development of hypotheses. The third section explains the methodology, including the research design and data collection methods. In the fourth section, the collected data is analyzed and interpreted. The fifth section focuses on discussing the findings derived from the analysis. Finally, the sixth and seventh sections conclude the paper by presenting the practical and theoretical implications of the research.

## Literature review

In technical terms, wearable payments permit consumers to make transactions on the move through wearable devices, which are almost analogous to the mobile payment terms^33^. The WPD conducts transactions through smart wearable devices that are physically attached to the users (e.g., smart-watches, rings, wristbands, RFID tags) for the purchase of products and services at any place and time^34^. Notably, WPD is one of the most recent technologies, such as the Apple Watch and digital rings, and is projected to eventually replace mobile and card payments^35^. When used in conjunction with an NFC-enabled device, the smart stamp allows users to perform card-less NFC payments^36^, such as MC10 and PCH. As a result, a Wearable Interactive Stamp Platform is created, which is a tattoo-like ultrathin stamp. Wearable contactless payment methods are gaining popularity due to their advanced technologies, which provide a quick, easy, and secure way to pay in various locations^34^.

### The unified theory of acceptance and use of technology (UTAUT)

The intention of using any new system is a prerequisite for the adoption of new technology. With the application of Technology Acceptance Model (TAM), Theory of Reasoned Action (TRA), and Theory of Planned Behaviour (TPB), several scholars investigated new technology usage intentions^37–39^. Upon the criticism of TAM, numerous modifications were presented in UTAUT model by Venkatesh et al.^30^. This model combined seceral technological acceptance and behavioural intention models, namely TAM, TPB, TRA and social cognitive theory^40^. According to UTAUT, four key determinants of behavioural intentions and actual adoption of every new technology include (i) efforts expectancy, (ii) performance expectancy, (iii) facilitating conditions, and (iv) social influence. Researchers have accepted UTAUT to examine the adoption behavior of new technologies, mainly in the context of organizational setups from its debut in 2003^41^. Other variables (age, gender, experience, and willingness to use) expected to moderate the impacts of predictors on usage intention and technology adoption^41^. According to Sobti^40^, the UTAUT model is able to explain 70% of the variation in the intention to use new technology. It was also argued that UTAUT is a more prominent model for recognizing the likelihood of success in introducing new technology and its determinants that impact users’ intention to use it^42^.

In the setting of movable device technologies, the Technology Acceptance Model, UTAUT, and UTAUT2 are the most commonly used models to measure consumers’ usage intentions^2,43^. Therefore, as an underpinning theory, UTAUT has found the platform from many previous research works, which successfully implemented UTAUT on several contactless payment technology usage intentions, such as m-payment^44^, NFC-based mobile payment^45^, mobile banking^42^, and e-wallet^3^. Furthermore, Williams^46^ performed a rigorous systematic review of previous research works that implemented the UTAUT model to build an understanding of the justification, advantages, influences, and limitations of the model. As a result, it was found that although most of the studies did not examine all the factors of UTAUT. Several other studies^3,45,47^ extended UTAUT and UTAUT2 with perceived trust as the factors affecting the adoption behaviour of contactless methods of payments. A few studies^3,32,48,49^ established lifestyle compatibility as one of the strong predictors of behavioural intention to use contactless payment technologies. Accordingly, this study responded to previous findings and recommendations, and extended two additional determinants, namely perceived trust, lifestyle compatibility, and UTAUT determinants, to empirically examine the effect of the determinants on intention and adoption of WPD in the Malaysian context.

### Development of hypothesis

#### Perceived usefulness (PU)

Perceived usefulness refers to the degree to which individuals believe that the adoption of a specific technology would improve their performance in activities^50^, which is similar to the performance expectancy under UTAUT^51,52^. The PU evaluates the intrinsic features of technology and how it could assist users in achieving task-based objectives, such as achieving high productivity in performing tasks^53^. To make a payment with WPD, users simply need to activate payment software on their smart wearable device and hold it to the terminal^1^ to reduce the long duration of transaction activities. Moreover, PU has been recorded as a crucial antecedent of payment-related intentions in different payment-related research works, such as FinTech^54^, mobile shopping^55^, mobile banking processes^56^, NFC payment methods^57^, m-payments^58^, and wearable payment processes^1^. Studies in various sectors, such as restaurant and hotel payments, revealed that PU was the most influential predictor for guests’ willingness to practice NFC-based payment systems^22,45^. Based on the study of wearable technology adoption in Malaysia by Chuah et al.^16^, it was found that PU was one of the most prominent predictors influencing users’ adoption intentions. To be specific, Lee et al.’s^1^ study on the intention to use WPD in Malaysia found that PU significantly and positively influenced users’ intention to adopt WPD. Based on previous study evidence, the following hypothesis was developed:

##### H_1_

PU possesses a positive influence on the intention to use WPD.

#### Perceived ease of use (PE)

Perceived ease of use determines the degree of consumers’ belief that the use of new technology requires little effort^50,59^. In this study, PE was identified as the perception of Malaysian youths, in which the effort would be minimal for acquiring and using wearable payment systems. Furthermore, PE has been adapted from Technology Acceptance Model (TAM), which is similar to Effort Expectancy in UTUAT^30^. Contactless payment systems, which take place by waving the user’s wrist, are simple and convenient as they allow customers to pay for purchased items simply with a movement of the hand^1^. The technology advancement contributed to an easier, faster, and safer process for users to perform contactless payments^3^. The research conducted by Shankar et al.^60^ identified PE as a crucial determinant to increase the intention of adopting contactless payment. Moreover, in the decrease in payment processing time, PE was considered an advantage for the adoption intention^51^. This result has been evident in numerous empirical studies, in which the PE in NFC-based payment was a robust predictor of consumers’ intention to adopt^61,62^. Ting et al.^63^ and Teo et al.^14^ also found that PE was a significant determinant of the Intention to use m-payment in the Malaysian context. As supported by earlier studies, the following hypothesis was proposed:

##### H_2_

PE possesses a positive influence on the intention to use WPD.

#### Social influence (SI)

SI is regarded as the value that customers place on the opinions of their close relatives in the decision of whether to use the new technology or vice versa^30^. Furthermore, SI indicates the inspiration from external factors on user’s behaviour, such as the experiences and views of close relatives, superiors, and friends^64,65^. It has also been argued that SI is the extent of social pressure applied on users to embrace new technologies^66^. Karjaluoto et al.^51^ stated that the social exchange of views and values significantly enhance perceived connecting capability and information adoption for new technologies to gain perceived benefits. Confirming the significance of SI, Oliveira et al.^67^ postulated that the adoption intention of m-payment is highly influenced by the recommendations and opinions of individuals who are prominent and famous in society. In Lu et al.’s^68^ investigation on 323 m-commerce users in the United States, SI was recorded as the most influential factor that stimulates consumers’ intention to continue using the service. Another study with 373 users of Mobile Money in Ghana revealed that higher SI would increase the users’ intention to adopt it^69^. Analysing all the previous studies on contactless payment technologies, the following hypothesis was proposed in this study:

##### H_3_

SI possesses a positive influence on the intention to use WPD.

#### Lifestyle compatibility (CM)

Technology is perceived as compatible in reducing the risk of using new technology with the values, experiences, and preferences of users^70,71^. Customers may regard new technology for payment as more compatible when they identify the advantages of using it for particular activities^67^ and the natural alignment of lifestyle choices^72^. Furthermore, CM has been identified to be a robust predictor of an individual’s behavioural intention in the adoption of new technologies^49^. Yang et al.^3^ discovered that CM possesses a significant relationship with users’ intentions to use digital payment. According to a study by Sitorus et al.^73^, the more compatible the mobile banking system is with the users’ values, habits, and lifestyle, the possibility for them to use it is higher. Herrero et al.^74^ found that CM is connected to prior lifestyle experiences and possesses a direct influence on the adoption behaviour of e-wallet. The finding of a study by Oliveira et al.^67^ demonstrated that the intention to utilise m-payment technologies was higher when the technology was compatible with the customers’ lifestyles and surroundings. Pham and Ho^75^ found that product-related factors (e.g., perceived utility, compatibility) had a compelling influence on the desire to adopt NFC-based payments. Overall, all the previous study results regarding the contactless payment methods led to the following hypothesis:

##### H_4_

CM possesses a positive influence on the intention to use WPD.

#### Perceived trust (TR)

Individuals’ feelings of willingness, security, and confidence to rely on a system and regularly fulfil their expectations to prevent failure are known as trust^76,77^. The TR plays a critical role in predicting one’s acquisition intention by minimising apparent risks throughout the transactions^78^. Individuals’ trust is crucial as it helps reduce customers’ worries, fears, and uncertainties, and increases adoption intentions^77^. The related security concerns for contactless payment may be addressed through trust-building approaches, such as the establishment of certifying authorities^23^. Trust plays a crucial role in inspiring customers to adopt contactless payment and constructing their viewpoint, which regards this technology as more creative and unique^56^. In an extended UTAUT study based on 296 experienced m-wallet users, Shin^79^ revealed that TR and security were two core factors to determine behavioural intention. In Yu et al.’s^80^ assessment of 219 Chinese m-payment customers, it was found that TR and innovation were two of the few determinants influencing the continued intention. The recent study by Yang et al.^3^ based on e-wallet usage intention among Indonesians recorded that TR had a strong and positive influence on the intentions to use WPD. Based on the evidence of contactless payment technologies, the following hypothesis was proposed:

##### H_5_

TR possesses a positive influence on the intention to use WPD (IWP).

#### Facilitating conditions (FC)

Facilitating condition denotes individuals’ belief towards technical infrastructures, which exist to facilitate the operation of a whole system^30^. The FC measures the extent of individuals’ consideration regarding the technical, financial, and related infrastructural resources that show adequate performance to support a new technology^81^. In UTAUT, FC has been defined as customers’ perceptions regarding the supports and resources available to utilise new technologies, which are believed to have a direct positive influence on the actual usage^41^. Consumers showed unwillingness to use mobile money upon discovering the lack of necessary operational assistance and financial resources^69^. Another study that included 342 m-payment users in Taiwan^82^ discovered that consumer adoption of the m-payment system was more favourable with better resource sufficiency, higher degree of support, and lower financial cost. In contrast, a study by Oliveira et al.^67^ based on the actual usage of m-payments among 301 students of universities in Portugal revealed that FC was not a crucial predictor for the intention and adoption of m-payment. Supporting Oliveira’s findings, the same result was observed in the study regarding the use of e-wallet among Indonesians, confirming that FC is a non-significant factor of consumer adoption^3^. Despite the contradictory results, these studies about contactless payment offered the platform to examine FC among Malaysian users for investigating their adoption of WPD. Therefore, considering the previous study findings and the theoretical direction, the following hypothesis was developed for this study:

##### H_6_

FC possesses a positive effect on the adoption of WPD.

### Intention to use WPD (IWP) and adoption of WPD (AWP)

The concept behind UTAUT is that behavioural intention determines the actual usage or actual adoption^41^. Customers with higher behavioural intention for adopting any new technologies have higher possibility to be the ultimate user^83^. According to previous research, actual adoption is generally initiated through users’ intention and evaluation of new technologies^30,41,84^. A systematic review of earlier literature work was performed by Turner et al.^85^, which encompassed 79 empirical studies on the impact of behavioural intention on new information system consumption. As a result, it was found that the intent to use any new technology was positively connected with actual adoption.

The research findings by Penney et al.^69^ revealed that the ultimate usage of Mobile-Money was largely dependent on the consumers’ intention to adopt the system. Nikou et al.^86^ examined the users’ interface and cognitive feedback to anticipate the association between the intentions to use e-wallet, while the empirical results illustrated that intention had a major impact on the adoption of e-wallet. This association has been empirically proven in various online and mobile banking adoption research works^56,87^. In a recent empirical study with 1,165 users in Finland, Karjaluoto et al.^51^ identified that higher consumers’ intention increased the adoption of contactless payment methods. Overall, the evidence from previous studies allowed the platform to present the following hypothesis:

#### H_7_

Intention to use WPD possess a positive influence on the adoption of WPD.

### Mediating effect of intention to use WPD (IWP)

Earlier studies highlighted the effect of PU^1,16,54–58^, PE^1,3,24,61–63^, SI^51,67–69^, CM^3,67,73–75^, TR^3,56,79,80^, FC^3,41,67,69,82^ on IWP. Furthermore, studies also highlighted a positive effect of IWP on AWP^51,56,69,86,87^. As the earlier studies highlighted the effect of PU, PE, SI, CM, TR and FC on IWP and the effect of IWP on AWP, this study therefore presents the following hypothesis:

#### H_8_

Intention to use WPD mediates the effect of PU, PE, SI, CM, TR and FC on the adoption of WPD.

### Moderation of age and gender

Age is identified as an important element in technological acceptability^88^. The importance of extrinsic rewards (equal to perceived usefulness) would increase among younger users as they are faced with fewer issues in processing complex stimuli. On the other hand, social influence would take place, in which older consumers are more affected by the opinions of others^89^. The findings of the research by Chawla and Joshi^72^ demonstrated that compared to elderly users, younger users are more motivated to embrace new technologies due to effective usefulness, higher level of trust, subjective norms, and innovativeness of the technologies. Another research by Liébana et al.^33^ proved that the impact of PU on the intention of using the m-payment system was comparatively higher amongst younger consumers compared to older users. In contrast, Liébana et al.^33^ study on m-payment adoption found no moderating effect of PU on the adoption intention of m-payments among younger users. The most recent study by Yang et al.^3^ illustrated that the age of users solely moderated the relationship between lifestyle compatibility and the intention of using e-wallets amongst Indonesian adults. Supported by the theoretical model and findings from earlier studies, the moderation impact of the age of Malaysian users was hypothesized as follows:

#### H_9_

Age of users moderates the effects of PU, PE, SI and FC on the intention to use WPD.

Based on social cognitive theories, the research by Kalinic et al.^90^ argued that men and women undergo different decision-making processes. To be specific, men concentrate on the results, performance, and usefulness, while women place more emphasis on security, privacy, and process orientation. According to Venkatesh et al.^59^, men are affected through perceived utility, while women are more inclined towards subjective norms and ease of use. Furthermore, Liébana et al.^33^ stated that men normally show more readiness to participate in e-commerce usage compared to women. Following that, the direct influence of usefulness on attitude among men is stronger compared to women. This condition implies that men have higher possibility to adopt a service upon their belief that it is beneficial and would assist them in achieving their goals^79^. Another study finding based on the m-payments context demonstrated that the impact of PU on the intention to use was significantly higher among men compared to women, while the influence of TR is substantially stronger among women compared to men^33^. The most recent study by Yang et al.^3^ illustrated that gender moderated the associations of PU, PE, and CM with the intention to use e-wallets, specifically among Indonesian women. Overall, the evidence from earlier studies and the theoretical model led to the following hypothesis:

#### H_10_

Gender of the users moderates the effects of PU, PE, and SI on the intention to use WPD.

**Figure 1:**
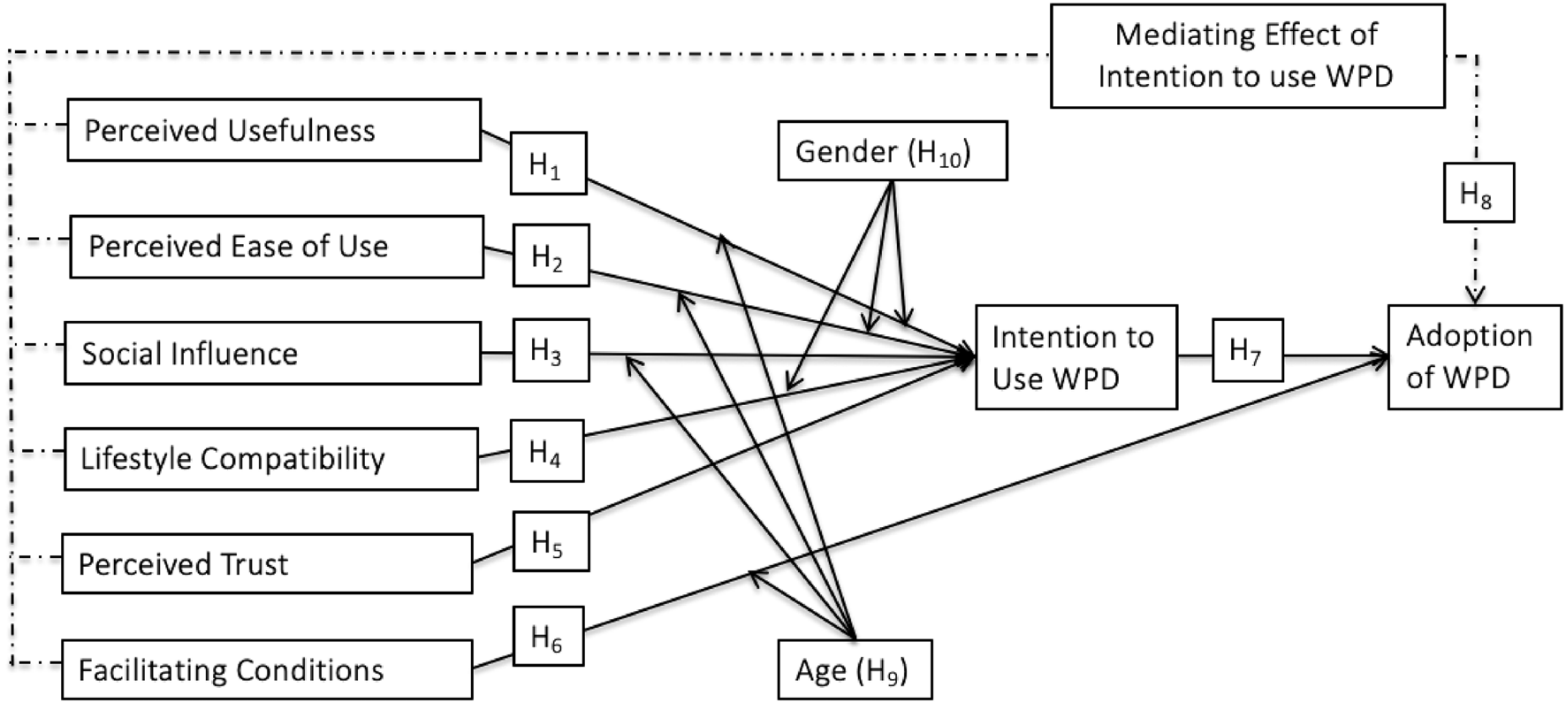
Conceptual framework.

All associations hypothesized are presented in Fig. 1.

## Research methodology

### Population and sample

This study adopted a cross-sectional design in the context of Malaysian youth to examine the adoption of WPD. The population of this study comprised 12.1 million people, while 37.12% of Malaysia's total population aged from 15 to 40 years old^91^. According to the International Labor Organisation^92^, 18–40 years old is categorised as youth and productive age, in which youths who are working adults from the formal sector obtain wage. Therefore, this study focused on Malaysian youths ageing from 15 to 40 years old, who were capable of earning and having the decision-making capability to become the adopters of wearable payment devices. G-Power was employed in this study to calculate the minimum sample using the effect size of 0.15, power of 0.80, and six predictors. Based on the estimation, the minimum sample size for this study was found to amount to 114 (source: https://webpower.psychstat.org/models/kurtosis/result). Data were collected randomly from the 1094 respondents to minimise the complications of a small sample size. The survey was performed online, followed by the collection of responses through a Google form. An online questionnaire link was distributed to the respondents via e-mail, Twitter, other online platforms, and through their peers from December 2020 to February 2021.

No formal ethics approval was required in this particular case because this research did not collect any medical information, there was no known risk involved, did not intend to publish personal information, and did not collect data from underaged respondents. This study has been performed in accordance with the Declaration of Helsinki. Written informed consent for participation was obtained from respondents who participated in the survey. For the respondents who participated in the survey online (using Google form), they were asked to read the ethical statement posted at the top of the form and proceed only if they agree. No data was collected from anyone under 18 years old.

### Measures of constructs

The survey questionnaire was adapted from previously tested and validated surveys, with minor changes included to fulfil the objectives of this study. Clear, understandable, and unbiased wordings were emphasized during the questionnaire development. To illustrate this point, respondents would find these features attractive and present earnest answers and reveal their thoughts. Five items were adapted from Lwoga and Lwoga^93^ and Chong et al.^94^ to measure perceived usefulness. Six items were derived from Karjaluoto et al.^51^ and Chawla and Joshi^72^ to test the perceived ease of use. To measure social influence, five items were adopted from Pandey and Chawla^95^ and Lwoga and Lwoga^93^. In the analysis of facilitating condition, this study implemented five items from Pandey and Chawla^95^, while four items were used to test lifestyle compatibility, which was extracted from Pandey and Chawla^95^. Six items were derived from Chong et al.^94^ and Chawla and Joshi^72^ to measure perceived trust. To test the intention to use, six items were adopted from Karjaluoto et al.^51^ and Chong et al.^94^. Apart from that, the items taken from Karjaluoto et al.^51^ for the confirmation of adoption of WPD through frequency of usage The survey questionnaire and sources were submitted as "supplementary material" together with this manuscript.

### Common method variance (CMV)

Common method variance is an impending problem in behavioural research^96^. The result of the CMV test for this study recorded the highest factor accounted for 44.619% of the variance, which was smaller than the threshold of 50%^96^. Therefore, it was proven that CMV was not a critical issue for the study data, and further analysis could be performed. This study also evaluated the CMV according to Kock’s^97^ recommendation to test the full collinearity (Table 1) for all the constructs. All the factors were retreated on the common variable, with the variance inflation factor values (VIF) found to be lower than 3.3. This result confirmed the nonexistence of bias from single-source data.

**Table 1:** Full collinearity test. Source: Author-generated data analysis.

PU	PE	SI	FC	CM	TR	IWPD	AWPD
2.388	2.820	2.345	2.576	2.894	2.354	2.398	1.231

PU: perceived usefulness; PE: perceived ease of use; SI: social influence; FC: facilitating conditions; CM: lifestyle compatibility; TR: perceived trust; IWP: intention to use wearable payment devices; AWP: adoption of wearable payment devices.

### Multivariate normality

Despite the fact that the PLS approach does not necessitate the multivariate normal distribution, Peng et al.^98^ cautioned against drawing broad generalisations about PLS capacity to estimate a model, given that it might contradict the standard of multivariate normality. Thus, Multivariate normality was tested through Web Power, an online tool that verifies data normality (https://webpower.psychstat.org/wiki/tools/index). Provided that multivariate coefficient *p*-values were below the threshold value of 0.05^99^, this test confirmed that the dataset was not normal.

### Method of data analysis

This study employed PLS-SEM to test the mediating role of the intention to use WPD between the relationships of all the constructs and adoption of WPD. The PLS-SEM has also been used to test the moderating effect of gender and age on the constructs. Furthermore, artificial neural network analysis has been deployed for a model-free estimation using parallel, multilayer, and non-linear regression. According to the standard practices of performing dual-stage analysis, PLS-SEM is initially used to determine the important exogenous factors, which are subsequently used as the input neurons for ANN analysis to thoroughly appreciate the non-linearity among the endogenous and exogenous factors^100^. As suggested by Hair et al.^101^, the statistical analysis for the dataset includes (a) descriptive analysis (mean and standard deviation), (b) internal reliability consistency with Cronbach’s alpha and Composite Reliability (CR) values that should exceed 0.7, (c) convergent validity (average variance extracted (AVE) that should be higher than or equal to 0.5^102^, (d) discriminant validity (loadings and cross-loadings) with the threshold value higher than 0.60^103^, and Heterotrait-Monotrait ratio (HTMT) and Fornell-Larcker criterion with the threshold value of less than 0.90^104^.

## Data analysis

### Demographic characteristics of respondents

Demographic characteristics for the study data are presented in Table 2. Within the total 1094 respondents in this study, the majority of the respondents were female (51.6%). Nearly half of the respondents (48.5%) aged below 21, while the second highest percentage (42.3%) aged between 21 and 25 years old. In terms of ethnicity, the majority percentage (82.2%) were Chinese. Furthermore, most of the respondents (94.3%) were single. More than half of the respondents (56.7%) have completed a Bachelor's degree or equivalent. In respect of the ratios of income status, the maximum percentage of respondents (80.7%) obtained a monthly income of less than RM2500. Apart from that, the majority of the respondents (88.1%) were from urban areas.

**Table 2:** Demographic characteristics. Source: Author-generated data analysis.

	n	%		n	%
*Gender*			*Education*		
Male	529	48.4	Secondary school certificate	192	17.6
Female	565	51.6	Diploma/technical school certificate	255	23.3
Total	1094	100.0	Bachelor degree or equivalent	620	56.7
*Age group*			Master degree or equivalent	21	1.9
			Doctoral degree	6	0.5
18–21 years	531	48.5	Total	1094	100.0
21–25 years	463	42.3	*Income*		
26–30 years	54	4.9			
31–35 years	18	1.6			
36–40 years	28	2.6	Less than RM 2500	883	80.7
Total	1094	100.0	RM 2500–RM 5000	150	13.7
*Ethnicity*			RM 5001–RM 7500	24	2.2
			RM 7501–RM 10,000	15	1.4
Malay	36	3.3	RM 10,001–RM 12,500	7	0.6
Chinese	899	82.2	More than RM 12,500	15	1.4
Indian	106	9.7	Total	1094	100.0
Others	53	4.8	*Living area*		
Total	1094	100.0			
*Marital status*					
Single	1032	94.3	Urban	964	88.1
Married	60	5.5	Rural	130	11.9
Divorced	1	0.1	Total	1094	100.0
Widowed	1	0.1			
Total	1094	100.0			

### Reliability and validity

The reliability and validity of the constructs were assessed, as shown in Table 3. In the Cronbach's Alpha (CA) reliability analysis, all of the constructs in this study exhibited a value of higher than 0.7, indicating that all the variables were reliable. Internal consistency reliability was also measured using Dijkstra-Hensele's *rho* (rho-A) and composite reliability (CR), which should have a threshold value of higher than 0.7^103^. Testing the internal CR, the results revealed that all the variables showed CR values higher than 0.891, indicating the high reliability of the constructs. Furthermore, AVE values were measured to reflect the total amount of variances in the observed variables accounted by the latent variables, which were relative to measurement errors^105^. Moreover, Fornell and Larcker^102^ specified that convergent validity should be assessed using AVE with the threshold value of higher than 0.50. The results for this study dataset indicated that all the items exhibited values of AVE ranging from 0.622 to 0.738, which were higher than the minimum suggested value of 0.50.

**Table 3:** Reliability and validity. Source: Author-generated data analysis.

Variables	Items	Mean	Standard deviation	Cronbach’s alpha	Dijkstra-Hensele’s *rho*	Composite reliability	Average variance extracted	Variance inflation factor
PU	5	4.038	0,082	0.848	0.851	0.891	0.622	2.318
PE	5	4.029	1.243	0.888	0.888	0.914	0.641	2.584
SI	5	3.436	1.144	0.86	0.863	0.899	0.64	1.866
FC	5	3.703	0.885	0.868	0.875	0.904	0.654	1.552
CM	4	3.819	0.852	0.876	0.877	0.915	0.728	2.894
TR	6	3.799	1.233	0.882	0.882	0.91	0.629	2.272
IWP	6	5.448	0.884	0.929	0.93	0.944	0.738	1.544
AWP	1	2.12	0.473	1	1	1	1	-

PU: perceived usefulness; PE: perceived ease of use; SI: social influence; FC: facilitating conditions; CM: lifestyle compatibility; TR: perceived trust; IWP: intention to use wearable payment devices; AWP: adoption of wearable payment devices.

Discriminant validity should be performed to investigate the relationship between the measures of possibly overlapped factors^106^. To obtain a more complex understanding and ensure the discriminant validity, this study employed three methods: Fornell-Larcker criterion, Heterotrait-Monotrait Ratio (HTMT) (Table 4)*,* and cross-loading (Table 5). Given that all the correlations between the constructs were found to be lower compared to the square root of the AVE, the conceptual model could be considered to have good discriminant validity^102^. HTMT is used to predict the discriminant validity of the factors with the threshold value equal to or less than 0.9^104^. All of the constructs in this study dataset were found to amount to 0.859 for Fornell-Larcker Criterion and 0.892 for HTMT, which met the threshold values. Besides the use of cross-loading for the comparison between the outer loadings of constructs, it was suggested in previous research works that the values for all factor loadings must exceed 0.60^103,107^. After testing the cross-loading for each item, the study dataset was found with all the factor loadings within the range of 0.744 to 1.00 and all positive values of higher than the suggested threshold value.

**Table 4:** Discriminant validity. Source: Author-generated data analysis.

	PU	PE	SI	FC	CM	TR	IWP	AWP
Fornell-Larcker criterion								
PU	0.788							
PE	0.697	0.801						
SI	0.512	0.516	0.800					
FC	0.596	0.669	0.721	0.809				
CM	0.668	0.673	0.645	0.774	0.853			
TR	0.579	0.655	0.573	0.642	0.683	0.793		
IWP	0.638	0.673	0.466	0.593	0.681	0.642	0.859	
AWP	0.286	0.243	0.384	0.385	0.334	0.248	0.278	1
Heterotrait-monotrait ratio (HTMT)								
PU	–							
PE	0.802	–						
SI	0.583	0.759	–					
FC	0.700	0.765	0.838	–				
CM	0.776	0.762	0.744	0.892	–			
TR	0.669	0.737	0.655	0.738	0.776	–		
IWP	0.716	0.740	0.516	0.663	0.753	0.706	–	
AWP	0.312	0.258	0.419	0.410	0.358	0.263	0.288	–

PU: perceived usefulness; PE: perceived ease of use; SI: social influence; FC: facilitating conditions; CM: lifestyle compatibility; TR: perceived trust; IWP: intention to use wearable payment devices; AWP: adoption of wearable payment devices.

**Table 5:** Loadings and cross-loading. Source: Author generated data analysis.

Items	PU	PE	SI	FC	CM	TR	IWP	AWP
PU1	0.838	0.572	0.410	0.486	0.563	0.457	0.550	0.255
PU2	0.785	0.509	0.443	0.501	0.539	0.456	0.459	0.272
PU3	0.765	0.528	0.435	0.482	0.528	0.463	0.466	0.225
PU4	0.785	0.578	0.353	0.447	0.517	0.463	0.523	0.199
PU5	0.767	0.557	0.385	0.439	0.487	0.444	0.507	0.180
PE1	0.529	0.777	0.340	0.485	0.485	0.463	0.483	0.171
PE2	0.560	0.826	0.440	0.571	0.569	0.549	0.547	0.184
PE3	0.588	0.814	0.542	0.434	0.556	0.535	0.554	0.211
PE4	0.545	0.825	0.452	0.556	0.545	0.549	0.538	0.221
PE5	0.556	0.813	0.415	0.553	0.547	0.547	0.549	0.205
PE6	0.567	0.744	0.387	0.500	0.524	0.496	0.554	0.173
SI1	0.434	0.468	0.776	0.519	0.459	0.458	0.406	0.265
SI2	0.435	0.426	0.823	0.543	0.514	0.506	0.389	0.218
SI3	0.405	0.421	0.815	0.613	0.560	0.454	0.388	0.345
SI4	0.400	0.376	0.803	0.610	0.548	0.431	0.354	0.371
SI5	0.362	0.354	0.781	0.611	0.500	0.434	0.312	0.357
FC1	0.501	0.525	0.625	0.803	0.623	0.527	0.467	0.304
FC2	0.474	0.554	0.565	0.812	0.610	0.482	0.458	0.306
FC3	0.460	0.545	0.568	0.831	0.618	0.479	0.491	0.327
FC4	0.501	0.565	0.543	0.758	0.641	0.578	0.508	0.254
FC5	0.487	0.531	0.613	0.838	0.648	0.547	0.483	0.352
CM1	0.578	0.574	0.567	0.694	0.844	0.591	0.555	0.280
CM2	0.559	0.568	0.543	0.652	0.855	0.559	0.564	0.299
CM3	0.583	0.590	0.531	0.637	0.862	0.601	0.622	0.270
CM4	0.560	0.564	0.561	0.663	0.851	0.581	0.578	0.295
TR1	0.458	0.499	0.471	0.526	0.553	0.803	0.486	0.195
TR2	0.488	0.539	0.485	0.551	0.571	0.836	0.533	0.230
TR3	0.437	0.487	0.448	0.496	0.504	0.814	0.450	0.194
TR4	0.459	0.533	0.455	0.502	0.550	0.788	0.519	0.212
TR5	0.460	0.522	0.475	0.510	0.562	0.759	0.518	0.177
TR6	0.445	0.528	0.389	0.466	0.504	0.756	0.536	0.168
IWP1	0.553	0.602	0.405	0.518	0.600	0.600	0.830	0.231
IWP2	0.500	0.561	0.322	0.468	0.523	0.517	0.804	0.195
IWP3	0.533	0.573	0.359	0.490	0.570	0.530	0.879	0.226
IWP4	0.562	0.604	0.432	0.515	0.599	0.551	0.895	0.248
IWP5	0.574	0.578	0.420	0.521	0.590	0.558	0.886	0.263
IWP6	0.560	0.548	0.455	0.538	0.619	0.550	0.854	0.266
AWP	0.286	0.243	0.384	0.385	0.334	0.248	0.278	1.000

PU: perceived usefulness; PE: perceived ease of use; SI: social influence; FC: facilitating conditions; CM: lifestyle compatibility; TR: perceived trust; IWP: intention to use wearable payment devices; AWP: adoption of wearable payment devices.

### Path analysis

As shown in Table 6, the path coefficient analysis demonstrated that PU to IWP showed a coefficient value (β) of 0.178, which was positive with the p-value of 0.000. This value was also lower than the significance threshold of 0.05. It was indicated that the PU possessed a positive significant influence on Intention to use WPD and H_1_ was supported. On the other hand, PE to IWP illustrated a positive β-value (0.240) and a significant p-value (0.000), indicating that PE was positively and significantly associated with IWP, and H_2_ was also supported. Furthermore, a difference was observed in the analysis of SI to IWP, which showed a negative β-Value of − 0.066 and a p-value of 0.017. Therefore, it was indicated that SI was statistically significant although the effect was negative. For this reason, H_3_ was not supported by this result. In respect of the remaining two associations, namely CM with IWP and TR with IWP, both the p-values (0.000 and 0.000) were statistically significant with positive β-values (0.294 and 0.216, respectively). Thus, H_4_ and H_5_ were supported. Additionally, all the values of f^2^ (except SI) indicated moderate to strong effect size of the constructs on intention to use WPD. Besides, the value of Q^2^ (0.430), which was higher than the threshold value of 0.00, illustrated a large predictive relevance of all the constructs with the intention to use WPD.

**Table 6:** Path coefficient. Source: Author generated data analysis.

Hypo		Beta	CI-min	CI-max	*t*	*p*	*r*^2^	*f*^*2*^	Q^2^	Decision
Factors affecting intention to intention wearable payment										
H_1_	PU → IWP	0.178	0.118	0.242	4.872	0.000	0.157	0.033	0.430	Supported
H_2_	PE → IWP	0.240	0.185	0.299	6.908	0.000		0.054		Supported
H_3_	SI → IWP	− 0.066	− 0.119	− 0.014	2.123	0.017		0.006		Rejected
H_4_	CM → IWP	0.294	0.234	0.352	8.277	0.000		0.073		Supported
H_5_	TR → IWP	0.216	0.159	0.277	6.315	0.000		0.050		Supported
Factor effecting the adoption of WPD										
H_6_	FC → AWP	0.335	0.281	0.389	9.885	0.000	0.589	0.086	0.151	Supported
H_7_	IWP → AWP	0.076	0.025	0.127	2.392	0.009		0.004		Supported

PU: perceived usefulness; PE: perceived ease of use; SI: social influence; FC: facilitating conditions; CM: lifestyle compatibility; TR: perceived trust; IWP: intention to use wearable payment devices; AWP: adoption of wearable payment devices.

It was recorded from the association between FC to AWP and IWP to AWP that both the associations showed the significant p-values (0.000 and 0.009, respectively) with positive β-values (0.335 and 0.076, respectively), indicating that FC and the intention to use WPD had a significant positive impact on the ultimate adoption of the WPD among the Malaysian youths. From these analyses, H_6_ and H_7_ were supported. The f^2^ value of 0.086 for Facilitating Condition to Adoption of WPD demonstrated the significant effect of this construct. As a final point of analysis, the Q^2^ value of 0.151 (which was higher than 0) showed a strong predictive relevance of FC and IWP with AWP. Besides, the value of r^2^ (0.589) indicated that these predictors could explain a significant proportion, such as 58.9% of the variation in the adoption of WPD.

### Mediation and moderating effects

The assessment of PLS-SEM correlations should not solely focus on direct effects, but it should also emphasise the indirect and total effects, which are the consolidations of the direct and indirect impacts in the structural model^104^. The mediating effect analysis of the intention to use WPD is illustrated in Table 7. The analysis revealed that the intention to use WPD fully mediates the associations between all the predictors (PU, PE, SI, CM, and TR) and adoption of WPD. The Path coefficient and p-value (*β* = 0.013, *p* = 0.017) indicated a statistically significant mediating effect of IWP on PU and AWP. Similarly, all the path-coefficients and p-values were found to be below the threshold values, which proved that the mediating effect of IWP was statistically significant between PE and AWP, SI and AWP, CM and AWP, and TR and AWP. The only exception was shown in the path coefficient value of SI with a negative value, which demonstrated that SI was negatively but significantly associated with AWP through the mediation of IWP. This result was in line with the direct path analysis between SI and AWP.

**Table 7:** Mediating and moderating effects. Source: Author generated data analysis.

Hypothesis		Beta	CI-min	CI-max	*t*	*p*	Decision
Mediating effect of IWP							
H_8_	PU → IWP → AWP	0.013	0.004	0.025	2.135	0.017	Mediates
	PE → IWP → AWP	0.018	0.005	0.031	2.316	0.010	Mediates
	SI → IWP → AWP	− 0.005	− 0.010	0.000	1.747	0.041	Mediates
	CM → IWP → AWP	0.022	0.006	0.039	2.286	0.011	Mediates
	TR → IWP → AWP	0.016	0.005	0.027	2.362	0.009	Mediates
Moderating effect of age							
H_9_	PU → IWP	0.003	− 0.002	0.007	1.061	0.145	No moderation
	PE → IWP	0.000	− 0.004	0.003	0.014	0.495	No moderation
	SI → IWP	− 0.003	− 0.008	0.002	1.052	0.147	No moderation
	FC → AWP	0.021	− 0.016	0.061	0.877	0.190	No moderation
Moderating effect of gender							
H_10_	PU → IWP	0.003	− 0.001	0.009	1.081	0.140	No moderation
	PE → IWP	− 0.003	− 0.008	0.001	0.922	0.179	No moderation
	SI → IWP	0.001	− 0.003	0.005	0.314	0.377	No moderation

PU: perceived usefulness; PE: perceived ease of use; SI: social influence; FC: facilitating conditions; CM: lifestyle compatibility; TR: perceived trust; IWP: intention to use wearable payment devices; AWP: adoption of wearable payment devices.

Moderation is a situation where the relationship between two or more constructs is affected by the third variable^104^. The analysis for the moderating effects of gender and age are presented in Table 7 below, along with the mediating effects. It was found that neither age nor gender had any moderating effects on PU, PE, SI, and intention to adopt WPD. Similarly, there was no moderation between FC and the adoption of WPD among the Malaysian youths.

### Artificial neural network (ANN) analysis

ANN is a robust and adaptable model, which does not require multivariate assumptions to fulfil (e.g., homoscedasticity, normality, multicollinearity, and linearity) compared to other linear approaches^18^. Thus, ANN models are considered more accurate and precise compared to linear models^108^. This section of the analysis focuses on predictive accuracy, which is estimated with the data section in the training and testing of the data. Furthermore, the root mean square of error (RMSE) values for training and testing (Table 8) of the data describes the relative accuracy of the prediction^109^. The values of RMSE for the study were recorded within the range of 0.437–0.470 in the training sample and 0.412–0.464 in test data for Model-A, which indicated the small and close values with high accuracy and strong predictive power of the study model^18^. Similarly, the values of RMSE for Model-B (0.773–0.786 for the training part and 0.760–0.792 for the testing part) demonstrated high prediction accuracy of the data fitness.

**Table 8:** Values of RMSE in artificial neural networks (N = 1094). Source: Author’s data analysis.

Network	Sample size (training)	Sample size (testing)	RMSE (training)	RMSE (testing)	Sample size (training)	Sample size (testing)	RMSE (training)	RMSE (testing)
	Model A: Factors affecting the intention to use the wearable payment device				Model B: Factors affecting the adoption of wearable payment device			
1	759	335	0.461	0.464	759	335	0.669	0.660
2	753	341	0.460	0.421	753	341	0.660	0.659
3	770	324	0.464	0.412	770	324	0.653	0.671
4	758	336	0.443	0.430	758	336	0.648	0.676
5	766	328	0.454	0.462	766	328	0.653	0.650
6	764	330	0.462	0.428	764	330	0.652	0.667
7	770	324	0.470	0.422	770	324	0.659	0.649
8	777	317	0.460	0.413	777	317	0.655	0.660
9	771	323	0.453	0.428	771	323	0.659	0.666
10	766	328	0.437	0.448	766	328	0.655	0.649
Mean			0.456	0.433	Mean		0.656	0.661
Standard deviation			0.010	0.019	Standard deviation		0.006	0.010

Sensitivity analysis is utilised for the evaluation of the contribution of exogenous predictors for all endogenous constructs^109^. The findings presented in Table 9 proved that the most influential variable intention to use WPD was CM, followed by PE and TR. In the case of the adoption of WPD, FC was the most influential factor.

**Table 9:** Sensitivity analysis. Source: Author’s data analysis.

Network	PU	PE	SI	CM	TR	FC	IWP
	Model A: Factors affecting the intention to use the wearable payment devices					Model B: Factors effecting the adoption of wearable payment devices	
1	0.172	0.291	0.098	0.318	0.121	0.963	0.037
2	0.242	0.26	0.094	0.186	0.218	0.671	0.329
3	0.186	0.147	0.039	0.314	0.314	0.881	0.119
4	0.194	0.275	0.057	0.198	0.276	0.872	0.128
5	0.181	0.25	0.07	0.258	0.241	0.810	0.190
6	0.214	0.222	0.059	0.287	0.218	0.902	0.098
7	0.204	0.234	0.068	0.277	0.218	0.947	0.053
8	0.217	0.209	0.041	0.338	0.196	0.903	0.097
9	0.253	0.176	0.045	0.253	0.272	0.655	0.345
10	0.271	0.251	0.081	0.201	0.196	0.940	0.060
Mean importance	0.213	0.232	0.065	0.263	0.227	0.854	0.146

PU: perceived usefulness; PE: perceived ease of use; SI: social influence; FC: facilitating conditions; CM: lifestyle compatibility; TR: perceived Trust; IWP: intention to use wearable payment devices.

## Discussion

Based on the result of the analysis, PU has been established as an important construct that could influence users’ intentions to adopt WPD. Numerous earlier literatures examined the impact of PU on the intention of using contactless payments, such as e-wallets and m-payments^3,18,57,110^ specifically for WPD^1^, which revealed similar outcomes. Furthermore, the customer’s standpoint was revealed, in which smart wearable devices could increase their work performance by combining many activities and multiple gadgets into a single device^8^. The result also highlighted the necessity of strengthening the consumers’ desire to use contactless payments by promoting more information about the advantages of it over the other systems to foster positive mindsets. Consequently, customers would perceive WPD as a convenient and smart method of making transactions. Furthermore, PE was recorded as the key factor to influence the intention to use WPD, which indicated that if WPD could be considered simple to use, customers had higher possibility to adopt it. The outcome was aligned with other earlier studies in the context of contactless payments and wearable devices^3,8,72^, although it was contradicted with the findings by Lee et al.^1^. The result of PE positive significant influence demonstrated that customers observed smart wearable devices and contactless payment. These methods comprise various appealing properties, which set these technologies apart from traditional payment methods, including the features of flexibility, mobility, efficiency, and customization^111^. Therefore, the respondents in this study perceived WPD as easier to use, with little effort made for paying for various products and services.

The study result analysis found that SI created a negative direction towards the intention of using WPD. Therefore, it was indicated that although SIs showed a statistically significant effect on the intention to use WPD, consumers showed negative reaction to the recommendations from their family and peers. Besides not supporting the theoretical direction, this result contradicted numerous previous studies of the behaviour towards the adoption of other contactless payment methods^3,18,56^. The study analysis revealed a positive and significant influence of lifestyle compatibility on the intention of using WPD. This result was analogous to the previous studies performed on other contactless payment technologies^3,67^. Moreover, it was indicated that consumers would adopt WPD if the technology is found to be well-matched with all phases of their lifestyle and well-fitted with their current standards of living^95^.

It was found that TR brought a significant and positive influence on users’ behavioural intentions to use WPD. A significant level of ambiguity was present in this payment mechanism among the respondents who were affected by TR due to the preliminary stage of the establishment of WPD in Malaysia. Furthermore, prior m-payment and e-wallet research revealed similar findings^3,18,110^, in which customers believed that the service was honest and reliable. Therefore, they preferred to use the service due to their high level of trust in it. Confidence in new technology contributed to enhanced assessments and attitudes, which were delivered upon their promises and trust in all safety features^3^. For this reason, consumers’ willingness to employ WPD was heavily influenced by level of confidence. Statistical result analysis empirically established the extensive influences of FC on the adoption of WPD. The result was parallel with the results in earlier studies on FC as the influencing determinant of the usage of m-banking^56,64^. However, the findings of FC impact contradicted the result by Yang et al.^3^ on e-wallet adoption behaviour, which confirmed that users placed significant interest in the availability of facilities, resources, supports, skills, and guidelines. These factors are essential to the effective and successful use of WPD. Clearly, the necessary facilities for operating wearable devices and contactless payments (e.g., smartphones, Internet access, 4G services, secured Wi-Fi, secured applications) are the basic features of performing smooth, flawless, and effortless transactions^56^. In a conclusion, consumers would likely adopt WPD if they were ensured with real-time support and assistance, financial and technological resources, access to software and hardware, and well-integrated and stable-service infrastructure that could guarantee all facilitating conditions.

Following the analysis of the mediating effect of intention to adopt WPD, this study showed a similar result with earlier study findings^3,112^. It was specified that the actual adoption of WPD was determined and controlled by users’ behavioural intention of using it. Therefore, users with a high level of intention would exhibit faster and easier adoption of WPD. However, gender and age showed no moderation on any constructs for the intention of using WPD in the Malaysian context, proving that men and women of all ages could equally adopt WPD technology. The potential reasons may be that the respondents are from a particular generation cohort (Gen Y), who are digital natives^113^ and have grown up with new technology. They use cell phones and other devices, along with social media, and have access to the internet. In terms of having no moderation of age, this study supported Yang et al.’s^3^ e-wallet adoption behaviour study findings, although it contradicted with the m-payment adoption behavioural studies by Chawla and Joshi^72^ and Liébana et al.^18^. Moreover, for gender moderation, this study did not support any of the findings regarding e-wallet and m-wallet adoption behaviour. This finding demonstrated that the determinants of the UTAUT model may not always exhibit similar impacts on the adoption intention of new technology and may vary in the country-to-country context.

## Theoretical and practical contribution

In terms of theoretical contribution, this study is one of the few studies that implemented UTAUT to investigate the intention of using WPD and the actual adoption of it in the context of Malaysia. The research model extended the UTUAT with additional two impelling determinants of intention to use WPD (perceived trust and lifestyle compatibility). However, the scarcity of research investigating these two factors in WPD was identified. One of the most noteworthy contributions of this study to the literature was present through the fact that it was founded on the outcomes of earlier studies performed on contactless payment methods, which introduced WPDs as a new form of payment. Another important contribution of this research to the existing literature was from its incorporation of a two-step analysis methodology (SEM-ANN), which was also deemed scarce in the studies of WPD employing UTAUT. Furthermore, ANN emphasised the influences of the most important independent variables (PE, TR, CM) and confirmed the high prediction accuracy of the data fitness through the analysis under a neural network. The result indicated that WPD should be architected and constructed with flexible, easier, and better features compared to all previous contactless payment systems, which are also functioning at present. For this reason, manufacturers should focus on simplifying the system to use with fewer complex options, allowing customers to make all payments at a fast rate to fulfil their purpose of making hassle-free transactions. At the same time, marketing practitioners should generate strategies that focus on the consumers’ CM, PU, and PE.

Given that perceived trust is a significant influencing factor in the intention to use WPD, the practitioners should identify what customers perceive as the hazards connected to the use of WPD in terms of trust^18^. To ensure a higher level of trustworthiness and privacy to users, payment providers should securely provide specific information about data privacy regulations, end-to-end encryption, biometric authentication facilities (e.g., fingerprint readers), secured networks, and sensor-based procedures among others. The direct association of facilitating conditions with the adoption of WPD was found to hold the highest importance in this study. Therefore, it was suggested that providers place the most focus on ensuring uninterruptable-service, well-structured backend system, good integration with other available payment methods, minimum downtime, well-managed data-backup, and available and easily understood information and guidelines among others to convince consumers that the system is stable and have better accessibility. Given that the analysis of this study revealed no moderation of age and gender, marketing practitioners should not only focus on young users as suggested by previous contactless payment system studies, but they also ought to explore the usage intentions of the elderly who gain revenue from indirect sources. This condition could broaden the range of pursuing potential consumers.

## Conclusion

The WPD technology remains at the nascent stage. A low number of studies were conducted to fully discover its potentiality in the sector of contactless payment technologies. While the use of wearable devices for payment is gaining traction, the wearable technology industry as a whole remains new and unstable^114^. This study was conducted with the view of providing insights into the intention of adopting this new technology as a fast and flexible payment method. Expanding the UTAUT by adding two determinants (perceived trust and lifestyle compatibility), this study has opened an opportunity for academics and scholars to conduct further investigation into other constructs for adopting WPD, such as perceived risks, perceived security, and perceived cost. Provided that the study adopted UTAUT for the investigation of Behavioural Intention, it has broadened the path of implementing other behavioural intention theories (e.g., DOI, TPB) to examine the innovation adoption for WPD.

Nevertheless, several limitations were present in this study. To be specific, cross-sectional design was used, which limited the controllability of unobserved heterogeneity that prevents a firm foundation for demonstrating causality. Future research works that apply longitudinal techniques should allow variable arrangements to be specified, constructed, and measured more effectively with the data collected over an extended period. Furthermore, the study was performed with a focus placed on young Malaysians who are advanced users of technology and financially stable. Thus, future studies should examine the model focusing on various demographic populations from a cross-country perspective, which comprises diverse cultural, environmental, economic, and technological circumstances to explore the behavioural intention of adopting WPD.

## Supplementary Information

Supplementary Information 1.

Supplementary Information 2.

## Data Availability

The original contributions presented in the study are included in the article/Supplementary Material, further inquiries can be directed to the corresponding author/s.
