# Supplementary material for: Predicting the intention and adoption of wearable payment devices using hybrid SEM-neural network analysis
Source: Sci Rep. 2023 Jul 11;13:11217. doi: 10.1038/s41598-023-38333-0 (PMC10336046; doi:10.1038/s41598-023-38333-0)
Supplement: Supplementary file 1 — Supplementary Information 1. [file 41598_2023_38333_MOESM1_ESM.docx]

**Supplementary Material 1.** Survey Questionnaires

| CODE | Items | Sources |
| --- | --- | --- |
| PU | Perceived usefulness | Lwoga and Lwoga (2017);  Chong et al. (2010) |
| PU1 | Using Wearable Payment Devices makes it easier for me to conduct my daily transactions |  |
| PU2 | Using Wearable Payment Devices allows me to manage my transactions more efficiently |  |
| PU3 | Using Wearable Payment Devices increases my productivity |  |
| PU4 | Using Wearable Payment Devices enables me to accomplish tasks e.g. payments more quickly |  |
| PU5 | Overall, I believe Wearable Payment Devices is more useful than traditional ways of conduct transactions |  |
| PE | Perceived ease of use | Karjaluoto et al. (2019);  Chawla and Joshi (2020) |
| PE1 | Learning how to use Wearable Payment Devices is easy for me |  |
| PE2 | My interaction with Wearable Payment Devices is clear and understandable |  |
| PE3 | I find Wearable Payment Devices easy to use |  |
| PE4 | It is easy for me to become skillful at using Wearable Payment Devices |  |
| PE5 | It is easy for me to remember how to perform task with Wearable Payment Devices |  |
| PE6 | I like the fact that payments done through Wearable Payment Devices require minimum effort |  |
| SI | Social influence | Lwoga and Lwoga (2017)  Pandey and Chawla (2019) |
| SI1 | People who influence my behavior think that I should use Wearable Payment Devices |  |
| SI2 | People who are important to me think that I should use Wearable Payment Devices |  |
| SI3 | Wearable Payment Devices are widely used by people in my community |  |
| SI4 | Almost all my friends use Wearable Payment Devices |  |
| SI5 | My family members use Wearable Payment Devices |  |
| FC | Facilitating Conditions | Pandey and Chawla (2019) |
| FC1 | I am given the necessary support and assistance to use Wearable Payment Devices |  |
| FC2 | I have the financial and technological resources required to use Wearable Payment Devices |  |
| FC3 | I have access to the software and hardware required to use Wearable Payment Devices |  |
| FC4 | The Wearable Payment Devices are well integrated and provided in a stable-service infrastructure |  |
| FC5 | My service provider/operator facilitates the use of Wearable Payment Devices |  |
| CM | Lifestyle Compatibility | Pandey and Chawla (2019) |
| CM1 | Using Wearable Payment Devices is compatible with all aspects of my lifestyle. |  |
| CM2 | Using Wearable Payment Devices fits into my lifestyle |  |
| CM3 | Using Wearable Payment Devices fits well with the way I like to purchase products and services |  |
| CM4 | Using Wearable Payment Devices is completely compatible with my current situation |  |
| TR | Perceived Trust | Chong et al. (2010)  Chawla and Joshi (2020) |
| TR1 | I trust that transaction conducted through Wearable Payment Devices is secure and private |  |
| TR2 | I trust payments made through Wearable Payment Devices channel will be processed securely |  |
| TR3 | I believe my personal information on Wearable Payment Devices will be kept confidential |  |
| TR4 | I believe wearable payment device providers keeps customers’ interests best in mind |  |
| TR5 | I believe that in case of any issue, Wearable Payment Devices service provider will provide me assistance |  |
| TR6 | I believe that the Wearable Payment Devices service providers follow consumer laws |  |
| IWP | Intention to use wearable payment device | Karjaluoto et al. (2019)  Chong et al. (2010) |
| IWP1 | Assuming that I have access to Wearable Payment Devices, I intent to use it |  |
| IWP2 | I intend to use Wearable Payment Devices if the cost and times is reasonable for me |  |
| IWP3 | I intend to use Wearable Payment Devices in the future |  |
| IWP4 | I intend to increase my use of the Wearable Payment Devices in the future |  |
| IWP5 | I intend to continue using Wearable Payment Devices more frequently in the future |  |
| IWP6 | I intend to use Wearable Payment Devices in my daily life |  |
| AWP | Adoption of wearable payment device | Karjaluoto et al. (2019) |
| AWP | On average, how often have you used Wearable Payment Devices per month? (Never, 1 to 5 times; 6 to 10 times; 11 to 15 times; More than 15 times) |  |
